# Multi-Metabolomics Coupled with Quantitative Descriptive Analysis Revealed Key Alterations in Phytochemical Composition and Sensory Qualities of Decaffeinated Green and Black Tea from the Same Fresh Leaves

**DOI:** 10.3390/foods11203269

**Published:** 2022-10-20

**Authors:** Jie Wang, Ying Zhang, Yan Liu, Shaorong Zhang, Linying Yuan, Yingfu Zhong, Xiuhong Wu, Juan Yang, Ze Xu

**Affiliations:** 1 Tea Research Institute, Chongqing Academy of Agricultural Sciences, Chongqing 402160, China; 2 College of Food Science, Southwest University, Chongqing 400715, China

**Keywords:** tea, caffeine, decaffeination, supercritical carbon dioxide, sensory quality

## Abstract

The supercritical CO_2_-based decaffeination (SCD) method can be used to prepare decaffeinated tea, but its overall effect on the phytochemicals, volatiles, and sensory qualities of green and black teas is still unclear, and its suitability to prepare decaffeinated green and black teas still needs to be compared. This study revealed the effect of SCD on phytochemicals, volatiles, and sensory qualities in black and green tea prepared from the same tea leaves, and compared the suitability of preparing decaffeinated green and black teas using SCD. The results showed that the SCD could remove 98.2 and 97.1% of the caffeine in green and black tea, respectively. However, it can cause further losses of phytochemicals in green and black teas, specifically the loss of epigallocatechin gallate, epigallocatechin, epicatechin gallate, and gallocatechin gallate in green tea and the loss of theanine and arginine in green and black teas. After the decaffeination, both green and black teas lost some volatiles but also generated new volatiles. Especially, the fruit/flower-like aroma, ocimene, linalyl acetate, geranyl acetate, and D-limonene, were generated in the decaffeinated black tea, while herbal/green-like aroma, β-cyclocitral, 2-ethylhexanol, and safranal, were generated in the decaffeinated green tea. The overall acceptance of decaffeinated green tea decreased due to the substantial reduction in bitterness and astringency, while the overall acceptance of decaffeinated black tea significantly increased. Therefore, SCD is more suitable for the preparation of decaffeinated black tea.

## 1. Introduction

Tea (*Camellia sinensis*) is a functional beverage that originated in Southwestern China. Its consumption is only second to water. According to the processing procedures and fermentation degrees, it can be classified into green, white, yellow, oolong, black, and dark tea [1]. Additionally, green and black teas have the highest consumption.

Tea contains various phytochemicals, including polyphenols, alkaloids, and amino acids, which contribute to sensory qualities and health-protecting functions. For example, catechins exhibit bitterness and astringency in tea infusion and have antioxidant, anti-tumor, and anti-inflammatory effects on the human body [2,3]. Theanine acts as a neuroprotector in the human brain and gives umami and sweetness to the infusion [4].

Caffeine has a refreshing effect and is an important bitterness contributor to tea infusion. However, excessive caffeine consumption may cause caffeinism, which symptoms include agitation, digestive disorders, and anxiety [5]. For caffeine-intolerant people, caffeine consumption may induce sleep disturbances (including poorer sleep and insomnia) and even cardiovascular problems [6,7]. Therefore, decaffeinated tea has recently been spotlighted.

Several factors affect the content of caffeine in decaffeinated tea, such as decaffeination technologies, tea types, and raw materials. Supercritical CO_2_ extraction is a technique for the separation and extraction of plant components using supercritical CO_2_ instead of conventional organic solvents. The fluid in a supercritical state has unusual phase equilibrium behavior and transfer properties with the solute in the mixture, and the solubility of the solute varies over a fairly wide range with changes in pressure and temperature, so this supercritical fluid can be used to extract specific components from a solid mixture [8,9]. The supercritical CO_2_-based decaffeination (SCD) method has been widely applicated to the preparation of decaffeinated tea. Sun, et al. [10] removed 70.2% of the caffeine in green tea using the supercritical CO_2_ extraction method. Still, Tang, et al. [11] used an ultrasonic-enhanced supercritical fluid method to remove 82.28% of the caffeine in the green tea. The SCD also removed other components in tea samples. Park, et al. [12] extracted 97.4% caffeine from the green tea with supercritical CO_2_ modified with 95% ethanol at 300 bar and 70 °C for 120 min, resulting in 62.2% loss of epigallocatechin gallate (EGCG). Huang, et al. [13] optimized the SCD for the extraction of caffeine using response surface methodology, and they extracted 95.6% caffeine from the green tea with supercritical CO_2_ and water modified at 300 bar and about 60 °C for 7.5 h, resulting in only 19.8% loss of catechins. From this, it is clear that the extraction of caffeine using SCD inevitably leads to the extraction of other substances, including catechins. Meanwhile, the SCD method may reduce the volatile compounds in green tea, which may affect the aroma qualities of green tea [14,15]. Thus, it is clear that the sensory qualities of teas could be changed after the extraction. However, the comprehensive alterations in phytochemical composition, volatile components, and sensory qualities of decaffeinated green and black teas are uncovered. In this study, green and black teas made from the same fresh leaves were used to prepare the corresponding decaffeinated teas to compare the effects of SCD on the phytochemical composition and sensory quality of black and green teas, and to compare the suitability of SCD in the preparation of decaffeinated green and black teas, using multi-metabolomics coupled with quantitative descriptive analysis.

## 2. Materials and Methods

### 2.1. Chemicals and Reagents

Chemical standards, including caffeine, phenolic acids, catechins, amino acids, and theaflavins were guaranteed reagents purchased from Sigma-Aldrich, Inc. (St. Louis, MO, USA). Other chemicals used were analytical grade and were purchased from Shanghai Titan Technology Co., Ltd. (Shanghai, China).

### 2.2. Processing and Decaffeination of Green and Black

The fresh leaves were harvested from the Yongchuan district, Chongqing, China. The same batch of fresh leaves was used to manufacture green and black teas. The green and black teas were processed using typical manufacturing approaches. Briefly, a portion of fresh leaves was fixed at 150–180 °C for 3 min to fixate the endogenous enzymes, followed by a rolling procedure for 50 min, and then dried at 95–110 °C for 5 min into the green tea. Another portion of fresh leaves was withered at 25 ± 2 °C and 70% humidity for 5 h and then rolled for 30 min. After that, the leaves were fermented at 28 ± 2 °C and 95% humidity for 3 h, followed by drying at 90–100 °C for 5 min to obtain the black tea. The obtained tea was stored at −80 °C until further use.

The tea leaves were slowly ground in a mortar. The particle sizes in the range of 0.3 to 0.6 mm in the tea samples were obtained using a sieve shaker. Then, the tea samples were decaffeinated using a supercritical CO_2_ extraction system (HA220-50-06, Nantong Huaan Supercritical Extraction Co. Ltd., Nantong, China). An amount of 500 g of tea samples was put into the extraction kettle, and then 150 mL of distilled water was pumped as the co-solvent into the extraction kettle. The system parameters were set as follows: extraction pressure, 32 MPa; temperature, 50 °C; time, 3.5 h; flow rate of CO_2_, 5 L/h. Next, the extraction system was opened, and then supercritical CO2 was introduced, and the extraction was completed after 3 h. The obtained tea leaves were dried in a vacuum dryer (SCIENTZ-30ND, Ningbo Scientz Biotechnology Co., Ltd., Ningbo, China) to obtain decaffeinated tea samples.

### 2.3. Total Water-Soluble Solid Content (TWSSC), Phenolics, Theaflavins, Theabrownins, Thearubigins, and Total Free Amino Acids (TFAAs)

TWSSC was measured using a previous method [16]. The tea was extracted twice at a tea-to-water ratio of 1 g/50 mL for 30 min at 80 °C. Then the extracts were combined and dried at 103 °C until constant weight. TWSSC of tea samples was expressed as mg/g DW. Total phenolics of tea samples were analyzed using the Folin–Ciocalteu method [17]. Briefly, 1 mL of 10% Folin–Ciocalteu reagent was added to 0.02 mL of the tea extract solution or water as a blank, followed by 0.18 mL of water. 0.8 mL of a 7.5% Na_2_CO_3_ solution was added after 5 min. Utilizing a Synergy H1MG microplate reader (BioTek Instruments Inc., Winooski, VT, USA), samples were incubated at 25 ± 2 °C for 60 min in the dark. The results were calculated using the gallic acid (GA) standard curve. Total theaflavins and thearubigins of tea samples were analyzed according to the previous method [18,19]. Three grams tea sample was extracted with 125 mL boiling water for 10 min and then immediately filtered to obtain the tea extract. The extract was mixed with 25 mL of ethyl acetate (EA) and then shaken for 5 min. The extract was allowed to stand for separation to obtain the EA extract and water extract. Mix 95% ethanol with 2 mL of EA extract until the total volume is 25 mL to obtain the Solution A. Mix 15 mL of EA extract with 15 mL of 2.5% NaHCO_3_ solution and shake rapidly for 30 s. After standing and layering, take 4 mL of EA extract and mix with 95% ethanol until the total volume is 25 mL to obtain the Solution C. Take 2 mL of the aqueous layer extract obtained from the first extraction and mix with 2 mL of saturated oxalic acid solution and 6 mL of water, then add 95% ethanol and mix until the total volume is 25 mL to obtain the Solution D. Mix 25 mL of tea extract with 25 mL of n-butanol and shake for 3 min. After separation, mix 2 mL of the aqueous layer with 2 mL of saturated oxalic acid solution and 6 mL of distilled water, then add 95% ethanol and mix until the total volume is 25 mL to obtain the Solution B. The absorbance *A* of each solution was measured at 380 nm by taking 200 µL of different solutions to be measured. Concentrations of theaflavins, thearubigins, and theabrownins were calculated using following equations:

$$\mathrm{Theaflavins}\left(\%\right)=\frac{A_{c}\times 2.25}{dried\;weight}\times 100\%\;\mathrm{Thearubigins}\left(\%\right)=\frac{\left(2A_{a}+2A_{d}-A_{c}-2A_{b}\right)\times 7.06}{dried\;weight}\times 100\%\;\mathrm{Theabrownins}\left(\%\right)=\frac{2A_{b}\times 7.06}{dried\;weight}\times 100\%$$

where *A_a_*, *A_b_*, *A_c_*, and *A_d_* are the absorbance of the solution A, B, C, and D, respectively.

TFAAs of tea samples were determined according to the ninhydrin colorimetric assay [20]. Briefly, 0.06 mL of the sample solution, standards, or water as a blank were mixed with 0.1 mL of phosphate buffer solution (pH 8), and then 0.06 mL of 2% ninhydrin was added. The mixtures were then heated in a water bath for 15 min. Prior to measurement at 620 nm, solutions were cooled to 25 ± 2 °C.

### 2.4. HPLC-Based Targeted Metabolomics

#### 2.4.1. Composition of Catechins and Caffeine

Catechins and caffeine were determined using an HPLC (Shimadzu Corporation, Kyoto, Japan) according to previous method with some modifications [20]. To prepare the test solution, the catechins and caffeine in the tea leaves were extracted using a ratio of 1:50 (g:mL, tea:70% methanol) in a water bath at 70 °C. The following were the chromatographic conditions: column: Agilent ZORBAX SB-C18 (5 µm, 4.6 × 250 mm); UV length: 278 nm; mobile phase (MP) A: 0.2% acetic acid (Ac) in water; MP: B, MeCN; rate: 0.9 mL/min; column temperature: 35 °C The gradient elution was shown in Table 1.

#### 2.4.2. Composition of Free Amino Acids

Free amino acids were determined using an HPLC [20]. In a water bath at 70 °C, free amino acids from tea were extracted using boiling water at a ratio of 1:50 (g:mL, tea: boiling water). DNFB was used to derivatize amino acids. The following were the chromatographic conditions: column: Shimadzu Inert Sustain AQ-C18 column (5 µm, 4.6 × 250 mm); UV length: 360 nm; MP A: NaAc (5 mM, pH 5.7)/THF (95:5, *v*/*v*); MP B: 80% MeOH; rate: 0.9 mL/min; column temperature: 35 °C. The gradient elution is shown in Table 2.

#### 2.4.3. Composition of Theaflavins

Theaflavins were determined using an HPLC [21]. In a water bath set at 70 °C, the theaflavins were extracted at a ratio of 1:50 (g:mL, tea:70% methanol). The following chromatographic conditions were used.: column: Agilent ZORBAX SB-C18 (5 µm, 4.6 × 250 mm); UV length: 278 nm; MP A: 2% Ac; MP B: MeCN: EA = 7:1 (volume ratio); rate: 0.5 mL/min; column temperature: 35 °C. The gradient elution is shown in Table 3.

#### 2.4.4. Composition of Organic Acids

Organic acids were determined using an HPLC [21]. At a ratio of 1:50 (g:mL, tea:70% methanol), organic acids were extracted from tea in a water bath at 70 °C. Chromatographic conditions were as follows: column: column: Sunfire C18 column (5 μm, 4.6 × 250 mm), UV detection at 210 nm; MP A: KH_2_PO_4_ (0.05 mM, pH= 2.7); MP B: 100% MeOH; rate: 0.5 mL/min; temperature: 30 °C; gradient elution: 5% B, 0–40 min.

### 2.5. GC-MS-Based Untargeted Metabolomics

With a few minor modifications, the previous study’s methods for collecting, identifying, and quantifying volatiles were used in this investigation [22]. The headspace solid-phase micro extraction-gas chromatography–mass spectrometry (Shimadzu Corporation, Kyoto, Japan) (HS-SPME-GC/MS) technique was used to investigate the volatile chemicals. For the extraction, 1 g of the tea sample and 5 mL of boiling water were put into a 10 mL vial. The internal standard solution was added with 5 L of the 25 g/mL ethyl caprate. The vial was quickly closed. When the vial was held in a water bath at 65 °C, the SPME fiber (50/30 m DVB/CAR/PDMS, Supelco, Bellefonte, PA, USA) was exposed to the headspace for 60 min. The SPME fiber was immediately placed into the GC/MS injector for thermal desorption. Under 230 °C injector temperature and splitless injection mode, the desorbed compounds were injected into a DB-5MS capillary column (30 m × 0.25 mm × 0.25 m, Shimadzu Corporation, Kyoto, Japan). Chromatographic conditions are shown in Table 4. The following MS parameters were used: 230 °C for the ion source, 40–400 *m*/*z* for the mass scan range, and 70 eV for the ionization energy.

### 2.6. Quantitative Descriptive Analysis

This investigation was completed by an expert group of 8 panelists from the Tea Research Institute at the Chongqing Academy of Agricultural Sciences (Chongqing, China). According to Xu, et al. [23], the sensory evaluations of the panelists for the flavor were graded on a 10-point scale. The sourness, sweetness, bitterness, umami, and astringency of the tea infusion were evaluated according to the intensities of citric acid, sucrose, caffeine, monosodium glutamate, and tannic acid as the taste references, respectively.

### 2.7. Color Parameters

A color measurement spectrophotometer, an Ultra Scan PRO (Hunter Associates Laboratory, Reston, VA, USA), was used to measure the tea infusions’ color properties, including L*, a*, and b* values.

### 2.8. Statistical Analysis

Each experiment was performed three times, and the data were presented as the mean ± standard deviation. Pearson’s correlation and principal component analysis (PCA) were analyzed. Data were analyzed by one-way analysis of variance to ascertain the significance. All statistical analyses were performed using SPSS Statistics software (Version 20, SPSS Inc., Chicago, IL, USA).

## 3. Results and Discussion

### 3.1. Alterations of Phytochemical Compositions of Green and Black Tea after the Decaffeination

#### 3.1.1. Non-Volatile Components

**Figure 1:**
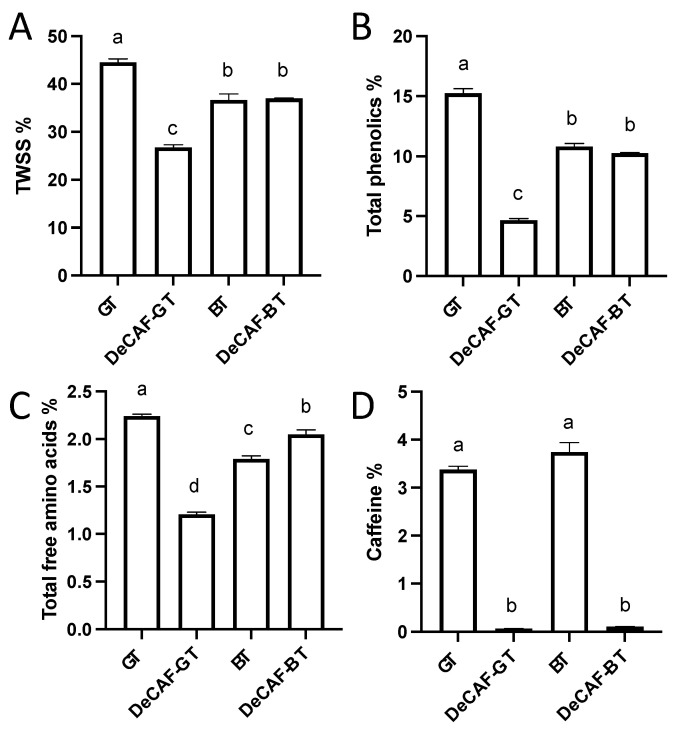
Contents of (**A**) total water-soluble solid (TWSS), (**B**) total phenolics, (**C**) total free amino acids, (**D**) caffeine of the green tea (GT), decaffeinated green tea (DeCAF-GT), black tea (BT), and decaffeinated black tea (DeCAF-BT). Different letters indicate significant differences (*p* < 0.05).

**Figure 2:**
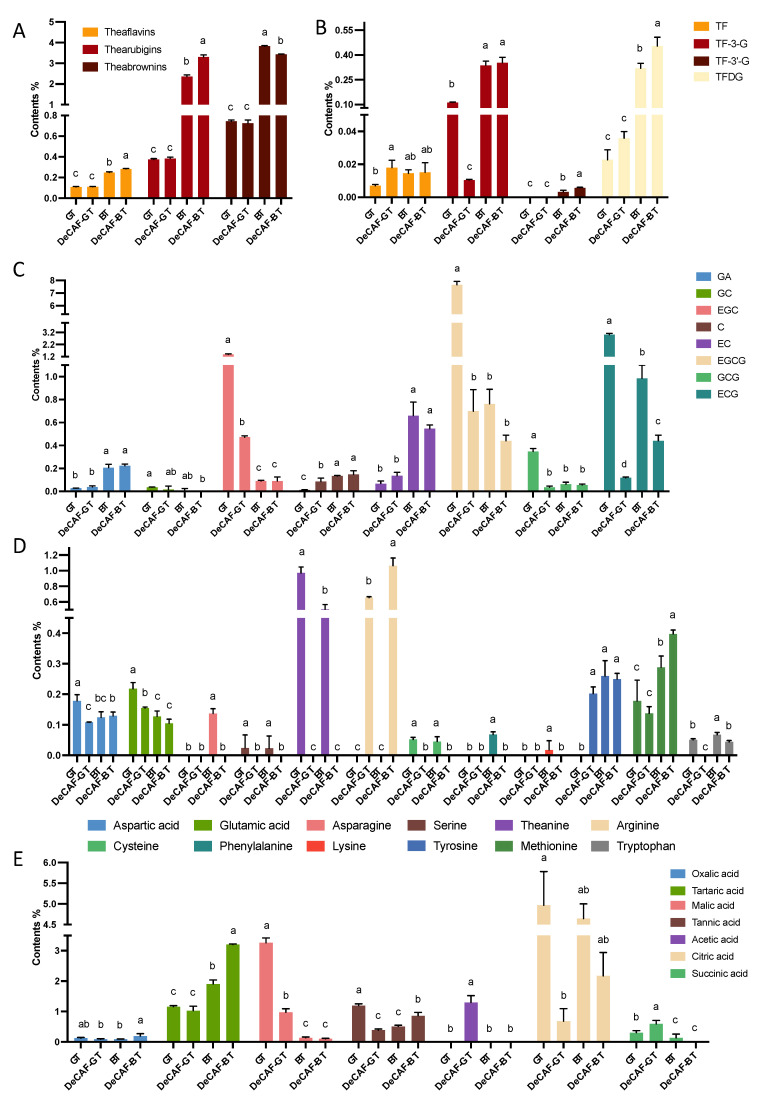
Contents of (**A**) total theaflavins, thearubigins, and theabrownins; (**B**) theaflavins, (**C**) catechins, (**D**) amino acids, and (**E**) organic acids of the green tea (GT), decaffeinated green tea (DeCAF-GT), black tea (BT), and decaffeinated black tea (DeCAF-BT). Abbreviations: theaflavins (TF); theaflavin-3-gallate (TF-3G); theaflavin 3’-O-gallate (TF-3’-G); theaflavin 3,3’-digallate (TFDG); epigallocatechin gallate (EGCG); gallocatechin (GC); epigallocatechin (EGC); catechin (**C**); epicatechin (EC); gallocatechin gallate (GCG); epicatechin gallate (ECG); gallic acid (GA). Different letters for the same component indicate significant differences (*p* < 0.05).

The green tea (GT) and black tea (BT) were made from the same fresh leaves. After their different processing procedure, the TWSS, total phenolics, and TFAAs of GT were significantly higher than that of BT (Figure 1A–C). However, the difference in caffeine content in these two samples was not significant, indicating that the effects of their processing procedures on caffeine do not significantly differ. These two samples were decaffeinated using the SCD method. The caffeine in GT and BT were dramatically reduced by 98.2 and 97.1%, respectively (Figure 1D). Although it’s effective for decaffeinating tea, the supercritical CO_2_ extraction method may remove other tea components and then affect the tea’s sensory qualities. In decaffeinated green tea (DeCAF-GT), the TWSS, TFAAs, and total phenolics were significantly lower than that in GT. However, the TWSS and total phenolics in BT and decaffeinated black tea (DeCAF-BT) were not significantly changed. In addition, the DeCAF-BT contained more TFAAs than that in BT. For the GT and DeCAF-GT, there were no significant differences in the contents of theaflavins, thearubigins, and theabrownins (Figure 2A). Compared with the BT, the decaffeinating technology led to higher theaflavins, higher thearubigins, and lower theabrownins in the DeCAF-BT. The polarity of thearubigins and theabrownins is not known, because of the lack of their exact chemical structure. However, it is clear that caffeine is more polar than theaflavins. Therefore, the solubility of theaflavins is lower than that of caffeine in a supercritical CO_2_ system with water as the co-solvent, and it has also been found in previous studies that the extraction rates of theaflavins, thearubigins, and theabrownins are considerably lower than those of caffeine in the same system [24]. Therefore, in this study, the loss of theaflavins, thearubigins, and theabrownins during the SCD should be considerably lower than that of caffeine. Although the degradation of theabrownins leads to the formation of theaflavins, thearubigins, and other components under high temperature and pressure are not clear, it’s believed that theabrownins are formed by polymerization of catechins, theaflavins, thearubigins, and others (e.g., polysaccharides and proteins) [25]. The higher theaflavins, higher thearubigins, and lower theabrownins in the DeCAF-BT may be contributed to the degradations of theabrownins under high temperature and pressure during the decaffeination processing, given that the degradation of theabrownins occurs under high temperature [26]. In addition, the total phenolics and TFAAs of DeCAF-BT were not lower than that of BT, which may be contributed to the degradation of theabrownins during the decaffeination processing. Meanwhile, after the SCD, the total weight of BT is reduced, which further contributes to an increase in the proportion of some substances (e.g., TFAAs, thearubigins, and theabrownins) in the tea leaves. Together, these may have contributed to the fact that the TFAAs and total phenolics in the DeCAF-BT were not significantly lower than that in the BT. However, it still needs further study to investigate why the TWSS was not significantly changed in DeCAF-BT. Phenolics and caffeine contribute to the bitterness and astringency of tea, and amino acids exhibit umami in tea [27]. For the BT, theaflavins, thearubigins, and theabrownins contribute to the characteristic flavor and color [28,29]. Thus, the decaffeination technology may significantly reduce the bitterness, astringency, and umami of GT and slightly reduce the bitterness and astringency of BT. This speculation will be verified in subsequent sensory experiments. Next, the phytochemical compositions in tea samples were analyzed in detail.

As is shown in Figure 2B, the theaflavin (TF) and theaflavin-3-gallate (TF-3G) were not significantly different between the BT and DeCAF-BT, but DeCAF-BT contained higher levels of theaflavin 3’-O-gallate (TF-3’-G) and theaflavin 3,3’-digallate (TFDG) than that of BT. After the decaffeination, the levels of GA, gallocatechin (GC), and epicatechin (EC) in the DeCAF-GT were not significantly changed, but the levels of epigallocatechin (EGC), epigallocatechin gallate (EGCG), gallocatechin gallate (GCG), and epicatechin gallate (ECG) were reduced by 66.2, 90.9, 88.6, and 96.0% in the DeCAF-GT, respectively. For the BT, all catechins (except for ECG) and GA changed insignificantly. The supercritical CO_2_ extraction method removed 87.2% catechins in the GT but only 39.3% catechins in the BT.

The SCD method also reduced most amino acids (aspartic acid, glutamic acid, serine, theanine, cysteine, and tryptophan) of the GT, and only levels of asparagine, phenylalanine, lysine, and methionine were not significantly changed. For the BT, asparagine, serine, theanine, cysteine, phenylalanine, lysine, and tryptophan were significantly reduced by the decaffeination process. Importantly, theanine has been regarded as an important umami-taste contributor in tea, and it was almost removed in the DeCAF-GT and DeCAF-BT, respectively. However, arginine levels increased to 0.656 and 1.06% in the DeCAF-GT and DeCAF-BT, respectively. Collectively, the SCD method removed 26.9% of amino acids in the GT but increased 18.5% of amino acids in the BT.

Organic acids are important flavor materials of sourness in tea. In the GT, acetic acid and succinic acid were significantly increased, and the decaffeination process significantly decreased malic acid, tannic acid, citric acid, and succinic acid. In the BT, only oxalic acid, tartaric acid, and tannic acid increased significantly after the decaffeination process, and other organic acids did not change significantly. Collectively, the SCD method removed 32.6% organic acids in the GT but increased 29.2% organic acids in the BT.

#### 3.1.2. Volatile Components

**Figure 3:**
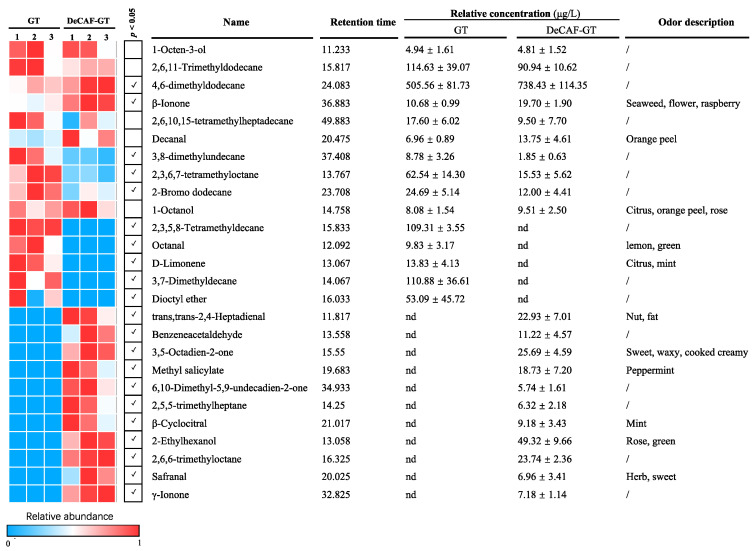
Non-volatiles in the green tea (GT), decaffeinated green tea (DeCAF-GT). Notes: the odor description was referenced from http://www.flavornet.org (accessed on 10 September 2022). The blue and red squares indicate the relative abundance of volatiles from 0 to 1, respectively. Abbreviations: not detected (nd).

**Figure 4:**
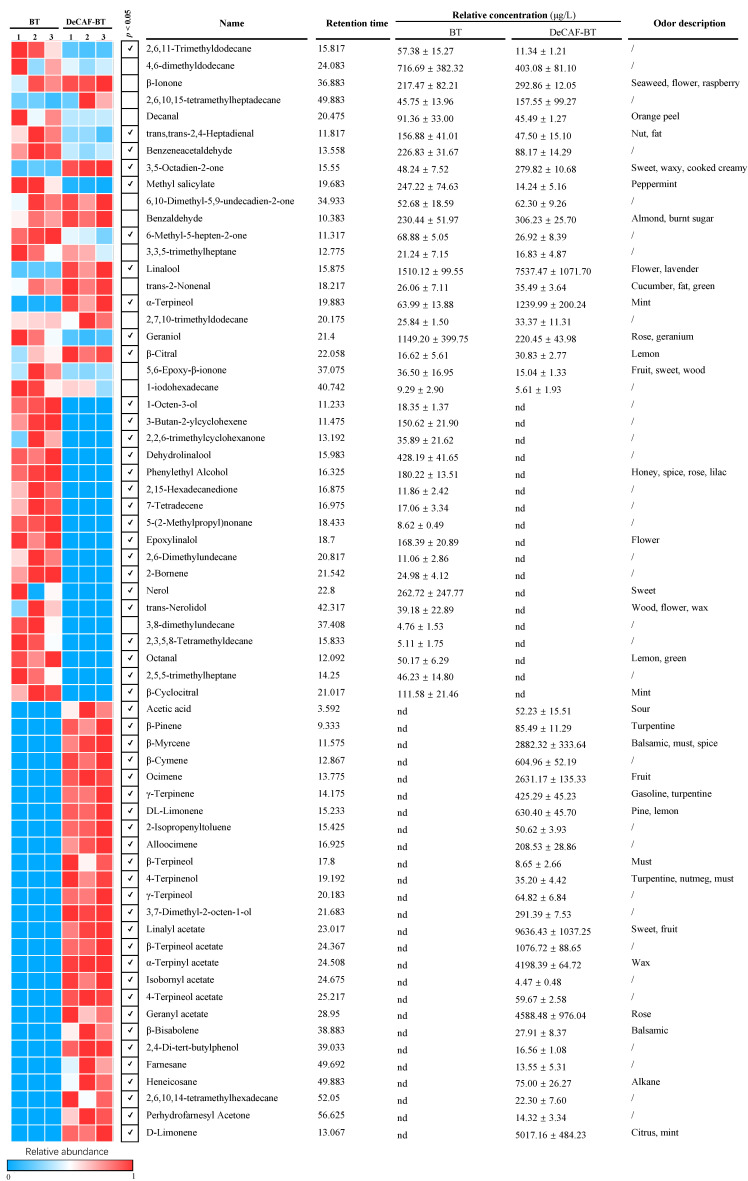
Non-volatiles in the black tea (BT) and decaffeinated black tea (DeCAF-BT). Notes: the odor description was referenced from http://www.flavornet.org (accessed on 10 September 2022). The blue and red squares indicate the relative abundance of volatiles from 0 to 1, respectively. Abbreviations: not detected (nd).

The changes in the volatile component in green and black tea samples were shown in Figure 3 and Figure 4, respectively. A total of 26 and 64 volatile compounds were identified in green and black teas, respectively. In the green tea, 4,6-dimethyldodecane and β-ionone were enriched in DeCAF-GT, whereas the decaffeination process decreased 3,8-dimethylundecane, 2,3,6,7-tetramethyloctane, and 2-bromo dodecane. Notably, five volatile compounds (e.g., octanal and D-limonene) were removed entirely in DeCAF-GT, but eleven volatile compounds were generated during the decaffeination process. Similar to our results, Joshi, Babu and Gulati [24] found that octanal and limonene were removed in green tea after the SCD. trans,trans-2,4-Heptadienal, methyl salicylate, β-cyclocitral, and safranal contributed to tea’s flower/fruit-like aroma, but they were hardly detected in green tea samples [30]. In our study, these volatile compounds were generated after decaffeination, indicating the potential aroma enrichment in DeCAF-GT. Lee, Park, Kim and Kim [14] studied the effect of supercritical carbon dioxide decaffeination on volatile components of green teas and found that 3-methyl-1-butanol, 3-methyl-2-pentanol, N,N-diethylbutanamine, and 4-methoxybutanoic acid were generated after the decaffeination process. Another study found that 2-ethyl-1-hexanol, (3Z)-3-hexen-1-yl hexanoate, and 2-pentylfuran were generated in green teas after the supercritical carbon dioxide decaffeination [31]. These results may be explained by the hydrolysis, volatilization, Maillard reaction, and redox reaction of aroma precursors, but the molecular mechanisms need to be revealed in further study [30]. The formation of β-cyclocitral and safranal may be due to the non-enzymatic degradation (auto-oxidation or thermal degradation) of carotenoids during tea processing [32]. In the BT, 2,6,11-trimethyldodecane, trans,trans-2,4-heptadienal, benzeneacetaldehyde, methyl salicylate, 6-methyl-5-hepten-2-one, and geraniol were lower in the DeCAF-BT, but 3,5-octadien-2-one, linalool, α-terpineol, and β-citral were increased after the decaffeination process. Of note, 17 volatile compounds were removed completely during the decaffeination process, whereas 26 volatile compounds were generated in the DeCAF-BT. 3,5-Octadien-2-one and α-terpineol exhibited sweet smelling and mint-like aroma, and they were enriched in the DeCAF-BT [33]. Linalool is a vital aroma contributor in black teas, which exhibits citrus-like and flowery aroma [34,35]. The decaffeination process significantly enhanced it, which may be explained by the degradation of trans,trans-2,4-heptadienal during the SCD [36]. The decaffeination process increased the levels of β-citral, which may contribute to the fruity-like aroma of the DeCAF-BT [34]. Acetic acid can provide a vinegar-like aroma and sour taste to tea infusion. In our study, the DeCAF-BT contained higher acetic acid than the BT, suggesting that DeCAF-BT may exhibit a stronger sour taste and strong vinegar-like aroma [33]. Ocimene and limonene, fruity-smelling compounds, were generated after the decaffeination. Similar to our study, Joshi, Babu and Gulati [24] found that γ-terpinene, farnesane, and cymene were generated in the Kangra orthodox black tea after the SCD. Conclusively, after the decaffeination processing, the changes of aroma substances in the BT were more severe than those in GT, indicating that the effect of decaffeination processing on the aroma of BT was stronger than that of GT.

### 3.2. Alterations of Sensory Qualities of Green and Black Tea after the Decaffeination

**Figure 5:**
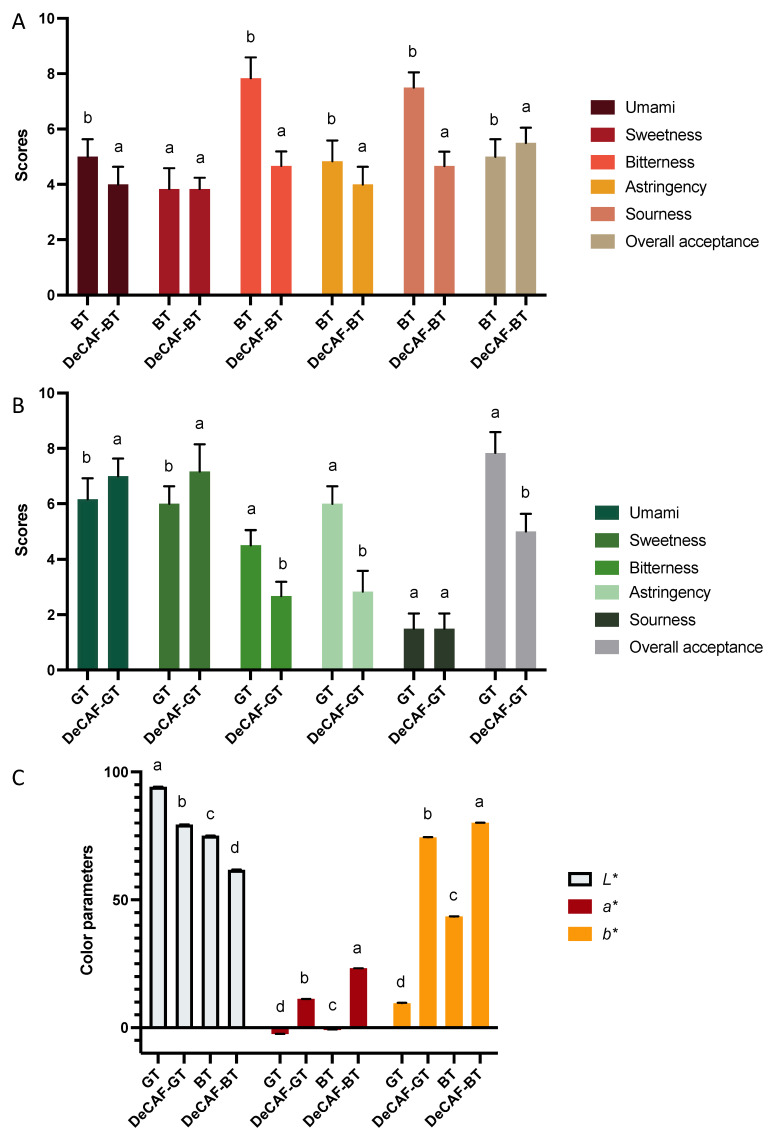
(**A**) The sensory qualities of black tea (BT) and decaffeinated black tea (DeCAF-BT). (**B**) The sensory attributes of green tea (GT), and decaffeinated green tea (DeCAF-GT). (**C**) The color parameters of the GT, DeCAF-GT, BT, and DeCAF-BT. Different letters for the same parameter indicate significant differences (*p* < 0.05).

The above studies have shown that decaffeinated processing significantly affects flavor compounds in BT and GT. Therefore, it is necessary to identify changes in the sensory quality of BT and GT through sensory evaluation and to compare the effect of decaffeination processing on the sensory quality of BT and GT. The overall acceptance was evaluated according to the taste and aroma of the tea infusion. Overall, the umami, bitterness, astringency, and sourness were decreased in the DeCAF-BT (Figure 5A). Although some bitter substances were increased (arginine and methionine) and some were decreased (caffeine, ECG, phenylalanine, and lysine) after decaffeination, the bitterness of BT infusion was reduced. These results may be explained by more bitter substances lost and the lower threshold of caffeine (97 μg/mL) and ECG (200 μg/mL), compared with that of arginine (13,065 μg/mL) and methionine (750 μg/mL) [20]. The reduction of the astringent substances ECG and theanine was consistent with the decline of astringency of the BT infusion after the decaffeination. Additionally, the reduction of umami in the DeCAF-BT infusion was consistent with the decrease in asparagine and theanine. However, the overall acceptance of the DeCAF-BT was significantly higher than the BT, indicating that the decaffeinated process helps to increase the sensory quality of the BT.

For the GT, the umami and sweetness were increased, but the bitterness and astringency were decreased after the decaffeination (Figure 5B). The reductions in bitter compounds (caffeine, EGCG, ECG, EGC, and GCG) and astringent compounds (EGCG, ECG, EGC, and theanine) in the DeCAF-GT are consistent with the changes in the bitterness and astringency of the DeCAF-GT infusion. Although the contributor of umami (asparagine and theanine) and sweetness (serine and theanine) decreased after decaffeination, the umami and sweetness of the DeCAF-GT were increased. These results may be explained by the lower bitterness and astringency reducing their impact on the perception of sweetness and umami in the tea infusion. Zhang, et al. [37] also reported that the strong taste from bitter and astringent components may lower the overall acceptance of GT.

The decaffeination exhibited similar impacts on the color parameters of the GT and BT infusion (Figure 5C). The values of L* decreased after the decaffeination, while the values of a* and b* were higher in the decaffeinated tea infusion. A negative a* value represents the green color, whereas a positive a* value represents the red color. The results are similar to the reports from Lin, et al. [38], who observed that the greenness of GT infusion became slight with lower levels of inner compounds.

### 3.3. Identification of Key Components That Affect Sensory Qualities of Tea Samples

#### 3.3.1. Pearson’s Correlation between Components and Sensory Qualities

Pearson’s correlation between phytochemicals and sensory quality in tea was analyzed to determine the key compounds affecting the quality of decaffeinated tea and to provide a reference for processing high-quality decaffeinated tea. As mentioned above, caffeine, EC, and lysine also highly impacted bitter taste, whereas EGC, EGCG, GCG, ECG, and theanine influenced astringent taste. These results were consistent with the Pearson’s correlation: bitterness versus lysine (r = 0.900), EC (r = 0.752), and caffeine (r = 0.719); astringency versus EGC (r = 0.590), EGCG (r = 0.790), GCG (r = 0.828), ECG (r = 0.921), theanine (r = 0.930). Overall acceptance was positively correlated with aspartic acid (r = 0.884), glutamic acid (r = 0.800), and theanine (r = 0.798), highlighting the positive contributions of umami and sweetness in overall acceptance. However, the overall acceptance was also positively correlated with EGC (r = 0.914), EGCG (r = 0.976), GCG (r = 0.980), ECG (r = 0.939). These results may be explained by the decaffeinated processing resulting in a substantial loss of bitter and astringent substances from the GT, resulting in a lower overall acceptance. Hence, to obtain a pleasant overall taste in decaffeinated tea, it is necessary to improve the processing conditions to reduce tea infusions’ bitterness and astringency.

#### 3.3.2. PCA

**Figure 6:**
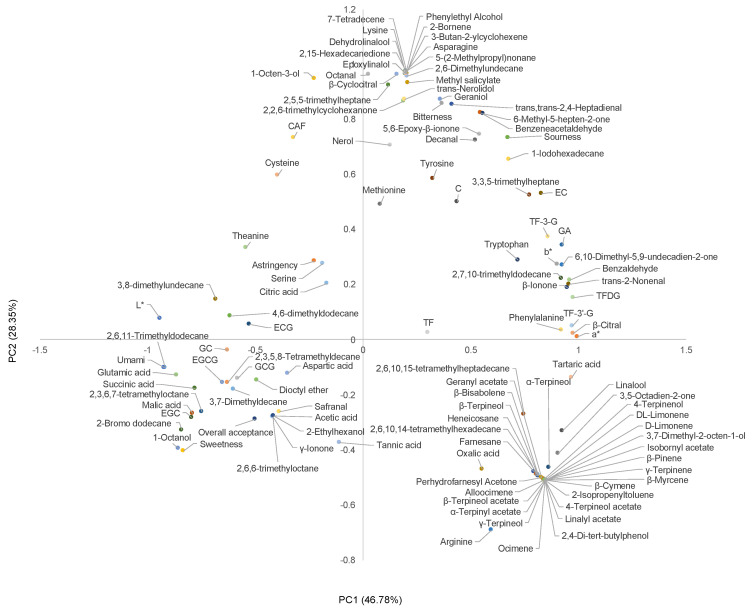
Principal component (PC) 1 vs. PC2 loading plots. Abbreviations: theaflavins (TF); theaflavin-3-gallate (TF-3G); theaflavin 3’-O-gallate (TF-3’-G); theaflavin 3,3’-digallate (TFDG); epigallocatechin gallate (EGCG); gallocatechin (GC); epigallocatechin (EGC); catechin (C); epicatechin (EC); gallocatechin gallate (GCG); epicatechin gallate (ECG); gallic acid (GA).

PCA was used to further analyze the correlation between phytochemicals and sensory profiles. Two PCs accounted for 75.13% of the original data on the variables. The loading values of variables in PC1 and PC2 are exhibited in Figure 6. For PC1, the overall acceptance was positively correlated with umami, sweetness, and astringency exhibited negative correlations with sourness and bitterness.

For PC1 and PC2, a* and b* values were positively correlated with TF, TF-3-G, TF-3′-G, and TFDG. The higher the a and b values, the more yellow and red the tea infusion is. For PC2, astringency was positively correlated with these theaflavins. It is reported that TF, TF-3-G, TF-3′-G, and TFDG are important yellow–red compounds in black tea, which exhibit astringency taste in black tea [21]. This is consistent with our results that theaflavins strongly contributed to the yellow-red and astringency of the black tea infusion.

In addition, umami exhibited positive correlations with glutamic acid and theanine, while astringency showed a positive correlation with tannic acid. This result was similar to previous studies in that glutamic acid and theanine were the main contributors to the umami of tea, and tannic acid strongly contributed to the astringency of the tea [27,39]. Previous studies have reported that geraniol and β-ionone can enhance the sweetness of tea infusion [21]. However, in our results, these two substances were negatively correlated with sweetness, indicating that the effects of geraniol and β-ionone on the sweetness of tea soups may are still limited. Safranal is a volatile substance with a sweet or herbal aroma; 1-octanol has a citrus aroma; 2-ethylhexanol exhibits a rose and green aroma, which is positively correlated with sweetness and overall acceptance, suggesting that it may contribute to the sweetness and overall acceptance of the tea infusion.

Collectively, for the GT, it is necessary to keep the bitter and astringent substances such as EC, EGCG, GCG, and ECG in the tea leaves at an appropriate level, during the decaffeination. Their high removal rate results in a bland and tasteless DeCAF-BT. For BT, theaflavins are the main contributors to the color and taste of the tea infusion. Their high retention rate is the key to maintaining the quality of DeCAF-BT.

## 4. Conclusions

In conclusion, SCD processing removes more than 97% of the caffeine in the GT and BT. However, the SCD processing leads to the loss of some key flavor compounds in the GT, such as catechins and amino acids. The SCD processing removed some of the volatile components in tea, but it also led to the formation of some volatile substances. After decaffeination, the types of volatile components in tea significantly increased. In terms of sensory quality, the decaffeinated processing resulted in a significant reduction in the bitterness and astringency of decaffeinated green tea, which negatively affected the overall acceptance of green tea. While the bitterness and astringency of black tea decreased, its overall acceptance increased significantly. So, the SCD processing is more suitable for the preparation of DeCAF-BT. Reducing the loss of bitter and astringent substances such as catechins during decaffeination may improve the sensory quality of GT.

## Figures and Tables

**Table 1:** The gradient elution of catechins and caffeine.

Time (min)	A (%)	B (%)
0	90	10
35	75	25
40	65	35
42	90	10
45	90	10

**Table 2:** The gradient elution of free amino acids.

Time (min)	A (%)	B (%)
0	90	10
20	80	20
25	50	50
34	45	55
50	0	100
55	90	10
60	90	10

**Table 3:** The gradient elution of theaflavins.

Time (min)	A (%)	B (%)
0	90	0
5	80	35
8	50	10
22	45	50
40	0	25

**Table 4:** The chromatographic conditions of volatiles.

Time (min)	Temperature (°C)
0	40
15	100
17	100
27	120
31	120
55	180
57	180
59.5	230
61.5	230

## Data Availability

Data is contained within the article.
